# Supplementary material for: EnzML: multi-label prediction of enzyme classes using InterPro signatures
Source: BMC Bioinformatics. 2012 Apr 25;13:61. doi: 10.1186/1471-2105-13-61 (PMC3483700; doi:10.1186/1471-2105-13-61)
Supplement: Addtional file 5 — The Java code to format the data files, evaluate and predict. The file enzml_java_code.tar.gz contains the Java code used to format database data to ARFF and XML formats, to execute cross and train-test (jackknife) evaluations and to record evaluation results to database. More information is included in the readme.txt file and the Javadoc files. The code can be used with a MySQL database. To use a different database software, other JDBC drivers might be required. [file 1471-2105-13-61-S5.gz › java_code/utils/doc/edu/cornell/lassp/houle/RngPack/RandomElement.html]

RandomElement


---


|  |  |  |
| --- | --- | --- |
| **Overview** \| **Package** \| **Class** \| **Use** \| **Tree** \| **Deprecated** \| **Index** \| **Help** | |  |
| **PREV CLASS**   **NEXT CLASS** | **FRAMES**    **NO FRAMES**     **All Classes** |  |
| SUMMARY: NESTED \| FIELD \| CONSTR \| METHOD | DETAIL: FIELD \| CONSTR \| METHOD |  |


---


## edu.cornell.lassp.houle.RngPack Class RandomElement

```
java.lang.Object
  cern.colt.PersistentObject
      edu.cornell.lassp.houle.RngPack.RandomElement
```

**All Implemented Interfaces:**: java.io.Serializable, java.lang.Cloneable

**Direct Known Subclasses:**: RandomJava, RandomSeedable, RandomShuffle

---

``` public abstract class RandomElement extends PersistentObject implements java.lang.Cloneable ```

RandomElement is an abstract class that encapsulates uniform random number
generators.

Subclasses have entirelly deterministic behaviour: Constructing two instances
with the same parameters at any two distinct points in time will produce
identical sequences of numbers.

To base a class on it, you must define the method `raw()` as
described below. It is also likely that you will want to define a constructor
or another mechanism for seeding the the generator. The other classes defined
in `RandomElement` add value to the numbers generated by
`raw()`

Source
code  is available.

**Version:**
:   1.0

**Author:**
:   Paul Houle  (E-mail:
    houle@msc.cornell.edu)

**See Also:**: `RandomJava`, `RandomShuffle`, Serialized Form

---

**Constructor Summary**

`RandomElement()`


| **Method Summary** | |
| --- | --- |
| `int` | `choose(int hi)` |
| `int` | `choose(int lo, int hi)` |
| `double` | `gaussian()`             gaussian() uses the Box-Muller algorithm to transform raw()'s into gaussian deviates. |
| `double` | `gaussian(double sd)` |
| `double` | `powlaw(double alpha, double cut)`             generate a power-law distribution with exponent `alpha` and lower cutoff `cut` |
| `abstract  double` | `raw()`             Returns a uniformly distributed pseudo-random number. |
| `void` | `raw(double[] d)`             Fill an entire array with doubles. |
| `void` | `raw(double[] d, int n)`             Fill part or all of an array with doubles. |
| `double` | `uniform(double lo, double hi)` |

**Methods inherited from class cern.colt.PersistentObject**

`clone`

**Methods inherited from class java.lang.Object**

`equals, getClass, hashCode, notify, notifyAll, toString, wait, wait, wait`

**Constructor Detail**

### RandomElement

```
public RandomElement()
```


**Method Detail**

### choose

```
public int choose(int hi)
```

:   **Parameters:**: `hi` - upper limit of range **Returns:**: a random integer in the range 1,2,... ,**hi**

---


### choose

```
public int choose(int lo,
                  int hi)
```

:   **Parameters:**: `lo` - lower limit of range: `hi` - upper limit of range **Returns:**: a random integer in the range **lo**, **lo**+1, ... ,**hi**

---


### gaussian

```
public double gaussian()
```

:   gaussian() uses the Box-Muller algorithm to transform raw()'s into
    gaussian deviates.

    :   **Returns:**: a random real with a gaussian distribution, standard deviation

---


### gaussian

```
public double gaussian(double sd)
```

:   **Parameters:**: `sd` - standard deviation **Returns:**: a gaussian distributed random real with standard deviation **sd**

---


### powlaw

```
public double powlaw(double alpha,
                     double cut)
```

:   generate a power-law distribution with exponent `alpha` and
    lower cutoff `cut`  

    :   **Parameters:**: `alpha` - the exponent: `cut` - the lower cutoff

---


### raw

```
public abstract double raw()
```

:   Returns a uniformly distributed pseudo-random number. The abstract method
    that must be defined to make a working RandomElement. See the class
    `RandomJava` for an example of how to do this.

    :   **Returns:**: a random double in the range (0,1). **See Also:**: `RandomJava`

---


### raw

```
public void raw(double[] d)
```

:   Fill an entire array with doubles. This method calls
    `raw(double d[],int n)` with `d=d.length`. Since
    this adds little overhead for `d.length` large, it is only
    necessary to override `raw(double d[],int n)`

    :   **Parameters:**: `d` - array to be filled with doubles.

---


### raw

```
public void raw(double[] d,
                int n)
```

:   Fill part or all of an array with doubles. The method defined here uses
    multiple calls to `raw()` to fill the array. You can eliminate
    the overhead of multiple method calls by subclassing this with a version
    of the generator that fills the array. On our system this improves the
    efficiency of `Ranecu` by 20% when filling large arrays.

    :   **Parameters:**: `d` - array to be filled with doubles: `n` - number of doubles to generate

---


### uniform

```
public double uniform(double lo,
                      double hi)
```

:   **Parameters:**: `lo` - lower limit of range: `hi` - upper limit of range **Returns:**: a uniform random double in the range (**lo**,**hi**).


---


|  |  |  |
| --- | --- | --- |
| **Overview** \| **Package** \| **Class** \| **Use** \| **Tree** \| **Deprecated** \| **Index** \| **Help** | |  |
| **PREV CLASS**   **NEXT CLASS** | **FRAMES**    **NO FRAMES**     **All Classes** |  |
| SUMMARY: NESTED \| FIELD \| CONSTR \| METHOD | DETAIL: FIELD \| CONSTR \| METHOD |  |


---
